# Investigating the Implications of a Variable RBE on Proton Dose Fractionation Across a Clinical Pencil Beam Scanned Spread-Out Bragg Peak

**DOI:** 10.1016/j.ijrobp.2016.02.029

**Published:** 2016-05-01

**Authors:** Thomas I. Marshall, Pankaj Chaudhary, Anna Michaelidesová, Jana Vachelová, Marie Davídková, Vladimir Vondráček, Giuseppe Schettino, Kevin M. Prise

**Affiliations:** ∗ Centre for Cancer Research and Cell Biology, Queen's University, Belfast, UK; † Department of Radiation Dosimetry, Nuclear Physics Institute CAS, Prague, Czech Republic; ‡ Proton Therapy Center Czech, Prague, Czech Republic; § Department of Dosimetry and Application of Ionizing Radiation, Faculty of Nuclear Sciences and Physical Engineering, Czech Technical University in Prague, Prague, Czech Republic; ‖ Radiation Dosimetry, National Physical Laboratory, Teddington, UK

## Abstract

**Purpose:**

To investigate the clinical implications of a variable relative biological effectiveness (RBE) on proton dose fractionation. Using acute exposures, the current clinical adoption of a generic, constant cell killing RBE has been shown to underestimate the effect of the sharp increase in linear energy transfer (LET) in the distal regions of the spread-out Bragg peak (SOBP). However, experimental data for the impact of dose fractionation in such scenarios are still limited.

**Methods and Materials:**

Human fibroblasts (AG01522) at 4 key depth positions on a clinical SOBP of maximum energy 219.65 MeV were subjected to various fractionation regimens with an interfraction period of 24 hours at Proton Therapy Center in Prague, Czech Republic. Cell killing RBE variations were measured using standard clonogenic assays and were further validated using Monte Carlo simulations and parameterized using a linear quadratic formalism.

**Results:**

Significant variations in the cell killing RBE for fractionated exposures along the proton dose profile were observed. RBE increased sharply toward the distal position, corresponding to a reduction in cell sparing effectiveness of fractionated proton exposures at higher LET. The effect was more pronounced at smaller doses per fraction. Experimental survival fractions were adequately predicted using a linear quadratic formalism assuming full repair between fractions. Data were also used to validate a parameterized variable RBE model based on linear α parameter response with LET that showed considerable deviations from clinically predicted isoeffective fractionation regimens.

**Conclusions:**

The RBE-weighted absorbed dose calculated using the clinically adopted generic RBE of 1.1 significantly underestimates the biological effective dose from variable RBE, particularly in fractionation regimens with low doses per fraction. Coupled with an increase in effective range in fractionated exposures, our study provides an RBE dataset that can be used by the modeling community for the optimization of fractionated proton therapy.

**Summary**

Clinical adoption of a constant cell killing relative biological effectiveness (RBE) for acute exposures underestimates the effect of increased linear energy transfer (LET) in the distal regions of clinical proton beams. Experimental data for the impact of dose fractionation in such scenarios remains limited. Toward distal regions of the spread-out Bragg peak, we found an increased RBE corresponding during fractionated proton exposures at higher LET and lower dose per fraction. This increase in RBE results in considerable deviation from clinically predicted isoeffective regimens.

## Introduction

Proton therapy is a rapidly advancing form of external beam radiation therapy and has been established as an alternative to photon-based modalities for specific cancer types (1). The motivation behind the adoption of therapeutic protons lies in their inherent physical advantages expressed over more conventionally used X-ray techniques. The inverted Bragg peak depth-dose profile allows for effective treatment of a tumor region while significantly sparing surrounding healthy tissues, with the superposition of several peaks of discrete energies treating extended regions using a spread-out Bragg peak (SOBP) 2, 3, 4. This increased sparing of healthy tissues offers additional advantages in the treatment of pediatric patients, in whom the risk of secondary cancers and late morbidity is significantly higher (5). Additionally, the rapid distal falloff in dose is ideal in the treatment of tumors located near organs at risk (OAR). Further to the advantages offered by the depth-dose profile, the increased linear energy transfer (LET) of protons in comparison with X-rays results in an increased biological effectiveness for cell killing. Particularly for regions surrounding the Bragg peak, the more localized pattern of energy deposition enhances biological damage, primarily through more complex DNA lesions 6, 7. To account for such an increase in effectiveness, the concept of relative biological effectiveness (RBE), defined as the ratio of photon to particle doses to induce an equivalent biological effect (8), is adopted in treatment planning to scale physical dose into RBE-weighted absorbed dose: D_RBE_, the product of physical dose and RBE 9, 10.

The estimation of proton beam RBE compared with energetic X-rays presents a key issue in radiation therapy because any uncertainty in the RBE transfers directly to an uncertainty in the biologically effective dose delivered to a patient. Considering the necessity of a 3.5% dose tolerance, characterization of proton RBE in a clinical setting is pertinent to the optimal delivery of proton radiation therapy (11). A lack of strong datasets has seen the clinical adoption of a fixed, generic RBE value of 1.1 regardless of evidence for changes in biological effectiveness as a function of energy modulation, beam size, cellular radiosensitivity, or SOBP size and position 12, 13, 14, 15. In addition, a fixed RBE during fractionated exposures disregards any effects resulting from the variation of dose per fraction and the total number of fractions delivered in relation to the LET (16).

As a key radiation therapy strategy, fractionation aims to maximize dose delivery to a treatment region while allowing healthy tissues time to repair by splitting the total dose into smaller fractions, with 1 or more rest periods between each delivery. Having reported a sharp rise in RBE toward the high LET regions of a clinical SOBP for acute exposures (12), the authors aim to elucidate the additional effects of a variable proton RBE on a fractionated regimen. The use of a variable RBE may see significant deviations from current clinical assumptions, and may obscure the potential therapeutic advantages of proton radiation therapy when delivering fractionated regimens based on the extrapolation of clinical experience with photons (17). The unique depth-dose deposition characteristics of protons may present opportunities for the shortening of clinical fractionation schedules through hypofractionation and have been investigated through several clinical trials 18, 19, 20. As a result, comparisons with the widely accepted International Atomic Energy Agency standard fractionation regimen of 2 Gy per fraction photon irradiation will provide useful insights for the discussion about the adoption of modified fractionation schemes (10).

For this study, the cell killing RBE of various proton fractionation regimens in normal human skin fibroblast (AG01522) cells were investigated. The pencil-beam scanning clinical beam of maximum energy 219.65 MeV at Proton Therapy Center, Prague, Czech Republic has been previously used in the treatment of a range of tumor sites including head and neck, brain, and prostate.

Cells were exposed at the positions of key features on a clinical dose profile: at the proton entrance and at the proximal-SOBP, central-SOBP, and distal-SOBP regions. These clinically relevant exposure conditions allowed the investigation of a wide range of clinical LET values. The effect of proton fractionation on cell survival was investigated by delivering up to 3 fractions to cell monolayers with an interfraction rest period of 24 hours.

## Methods and Materials

### Cell culture

AG01522 cells were maintained in α-modified minimum essential medium (Sigma Aldrich, St. Louis, MO) supplemented with 20% fetal bovine serum and 1% penicillin-streptomycin (Gibco, Life Technologies Carlsbad, CA). All cells were incubated in 5% CO_2_ with 95% humidity at 37°C.

### Proton irradiation, dosimetry, and simulation

Cells were exposed at various positions along a clinical SOBP generated by pencil scanning beam of maximum energy 219.65 MeV, generated by an IBA Protheus 230 cyclotron at Proton Therapy Center Prague, Czech Republic. Up to 3 fractions (of the same dose per fraction) were delivered to cells with an interfraction rest period of 24 hours. The full details are outlined in Supplementary Information (available online at www.redjournal.org).

### Clonogenic assay

Cells were incubated in full media for 24 hours before the delivery of each fraction. After the delivery of the final fraction, cells were immediately trypsinized, counted, and seeded onto 6-well plates in duplicate with sufficient density to obtain ∼50 macroscopic colonies per well. Plates were then incubated in 5% CO_2_ with 95% humidity at 37°C for 12 days to allow macroscopic colony formation. Colonies were fixed and stained using 0.5% crystal violet dye in 95% methanol in water for 30 minutes at room temperature, then gently rinsed in water and air dried. Colonies consisting of at least 50 cells were scored as viable.

### Data analysis and simulation

Cell survival was described using a linear quadratic formalism, where for acute exposures the surviving fraction (SF) of cells after receiving an acute dose D is given by:

(1) $$SF_{acute}=\text{exp}\left(-\;\alpha D-\;\beta D^{2}\right)$$

with fitting parameters *α* and *β*. Additionally, cell survival after a fractionated regimen of *n* fractions and dose per fraction *d* is described as follows:

(2) $$SF_{frac}=\text{exp}\left(-\;\alpha \;n\;d-\;\beta \;n\;d^{2}\right)$$

Using the definition of RBE calculated relative to 225 kVp X-rays (D_X rays_/D_protons_ at isoeffect where D denotes acute dose), it is possible to obtain analytic equations for the RBE as a function of the radiation dose in acute and fractionated regimens, where

(3) $${\text{RBE}}_{\text{acute}}=\frac{–\;\alpha_{X}+\sqrt{\alpha_{X}^{2}+\;4\;\beta_{X}\left(\alpha_{P}\;D_{P}\;+\;\beta_{P}\;D_{P}^{2}\right)}}{2\beta_{X}\;D_{P}}\;\;$$

and

(4) $${\text{RBE}}_{\text{frac}}=\frac{\alpha_{P}+\beta_{P}d_{P}}{\alpha_{X}+\;\beta_{X}\;d_{X}}\;\;$$

where *X* and *P* subscripts denote parameters corresponding to X-ray and proton exposures, respectively. Nonlinear regression analysis was performed on survival curves using GraphPad Prism version 6.0f. A detailed description of the simulation parameters and toolkit used is provided in Supplementary Information (available online at www.redjournal.org).

## Results

### Cell survival by fractionation regimen

**Fig. 1:**
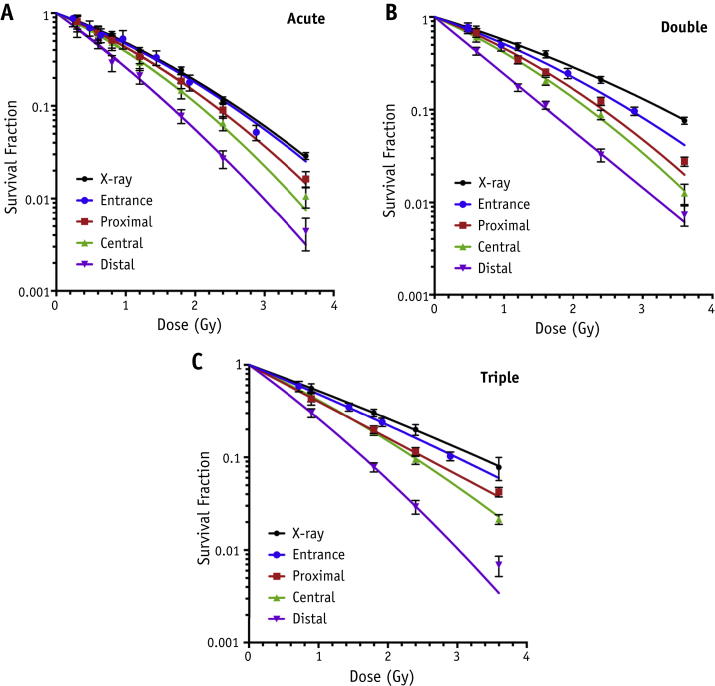
Clonogenic survival data at experimental positions in the entrance, proximal, central, and distal regions of the spread-out Bragg peak for AG01522 cells alongside reference 225 kV_p_ X-ray curves. Survival curves indicate overall cell survival after irradiated dose delivered under each fractionation regimen. (A) Cell survival as a function of total dose delivered in a single (A), double (B), and triple (C) exposure at the 4 experimental positions. Error bars indicate standard error of the mean with fits obtained using the linear quadratic model.

Figure 1 details the cell survival under the various proton fractionation regimens alongside reference X-ray survival curves. It is evident that for all fractionation regimens, cell survival curves become consistently steeper toward more distal positions and remain steeper than the X-ray curves in all cases. With the introduction of more fractions, the level of cell sparing increases across all positions but varies along the SOBP, with the most distal positions seeing the least amount of sparing. The fold decrease (ie, SF_distal_/SF_proximal_ at 3.6 Gy) in survival between the proximal and distal positions is 3.7 ± 1.0 and 3.8 ± 0.8 for the single-fraction and double-fraction regimens but is increased to 6.1 ± 1.3 for a triple-fraction regimen, where a total dose of 3.6 Gy is delivered.

### Cell survival by SOBP position

**Fig. 2:**
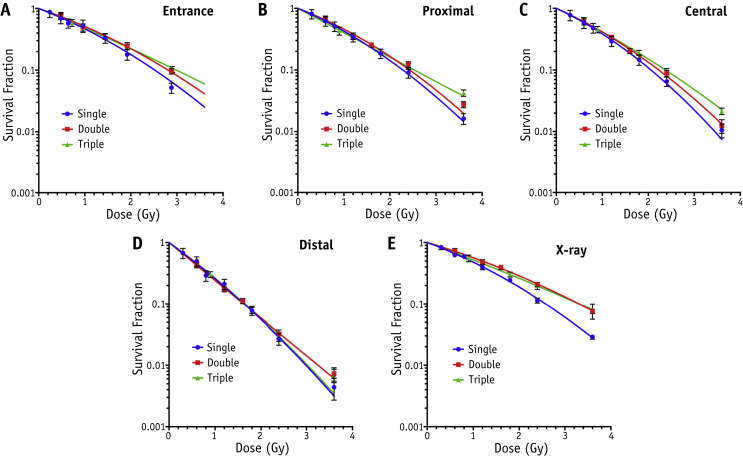
Clonogenic survival data under the 3 fractionation regimens delivering total dose in single, double, and triple fractions for AG01522 cells alongside reference 225 kV_p_ X-ray curves. Survival curves indicate overall cell survival at each experimental position at the entrance (A), proximal (B), central (C), and distal (D) regions of the SOBP with LET = 0.63, 1.68, 2.45, and 7.5 keV/μm, respectively. X-ray response is described in (E). Error bars indicate standard error of the mean with fits obtained using the linear quadratic model (full details outlined in Supplementary Information; available online at www.redjournal.org).

Figure 2 details the cell survival at the various experimental SOBP positions alongside reference X-ray survival curves. Again, in all cases the increased level of cell sparing with increasing number of fractions is evident: the fold increase in cell survival between triple-fraction and single-fraction regimens for the proximal and central positions at 3.6 Gy is 2.59 ± 0.27 and 2.0 ± 0.4, respectively. However, the effect of fractionation is less evident in the positions with higher LET, with cell survival curves effectively overlapping regardless of fractionation regimen at the distal position, with fold increase in cell survival of 1.6 ± 0.27 between single-fraction and triple-fraction regimens at 3.6 Gy.

### Cell survival by fraction size

**Fig. 3:**
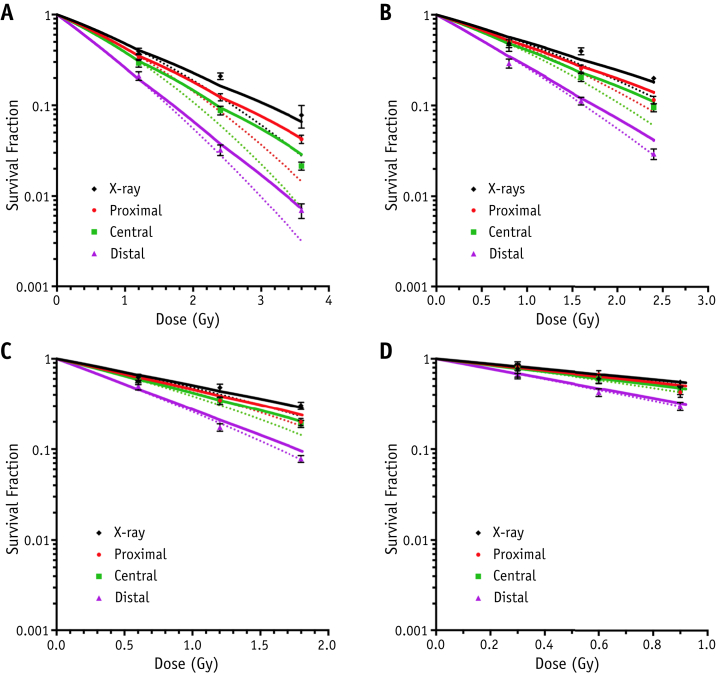
Clonogenic survival data at experimental positions in the proximal, central, and distal regions of the spread-out Bragg peak for AG01522 cells alongside reference 225 kV_p_ X-ray curves. Survival curves indicate cell survival for (A) 1.2 Gy, (B) 0.8 Gy, (C) 0.6 Gy, and (D) 0.3 Gy per fraction for up to 3 fractions. Error bars indicate standard error of the mean, solid lines represent fits obtained using the fractionated linear quadratic model (equation 2), and dotted lines are for the acute exposures.

Figure 3 shows survival data after delivering single, double. and triple fractions of 1.2, 0.8, 0.6, and 0.3 Gy per fraction alongside reference X-ray data. For all fraction sizes, fractionation of the proximal and central positions allowed significantly more cell sparing than the distal region, where survival curves were significantly steeper. Adoption of a linear quadratic formalism to predict fractionated response based on the cell response parameters of a single fraction appears suitable, matching experimental data points closely across all data sets. The comparison of experimental versus analytically obtained survival for single, double, and triple exposures yields Pearson's correlation coefficients >0.975 (*P*<.0001), indicating an excellent degree of correlation (Fig. E2; available online at www.redjournal.org).

### The clinical implications of a variable RBE under various fractionation regimens

A strong linear relationship of the proton α parameter α_p_ with LET (Fig. E3; available online at www.redjournal.org) allows the parameterization of RBE in acute and fractionated regimens by substituting the expression

(5) $$\alpha_{p}=\alpha_{x}+\lambda \text{LET}$$

in equations (3), (4), where α_p_ can be described in terms of the α parameter for X-ray exposure α_x_, proton LET and the linear gradient of the acute cell response (λ = 0.0883, characteristic for the cell line used). Inasmuch as no significant difference or relationship between proton β parameters β_p_ and LET was observed, the parameterized β values were assumed to be constant and equivalent to those for the X-ray response.This is in agreement with published work (21).

**Fig. 4:**
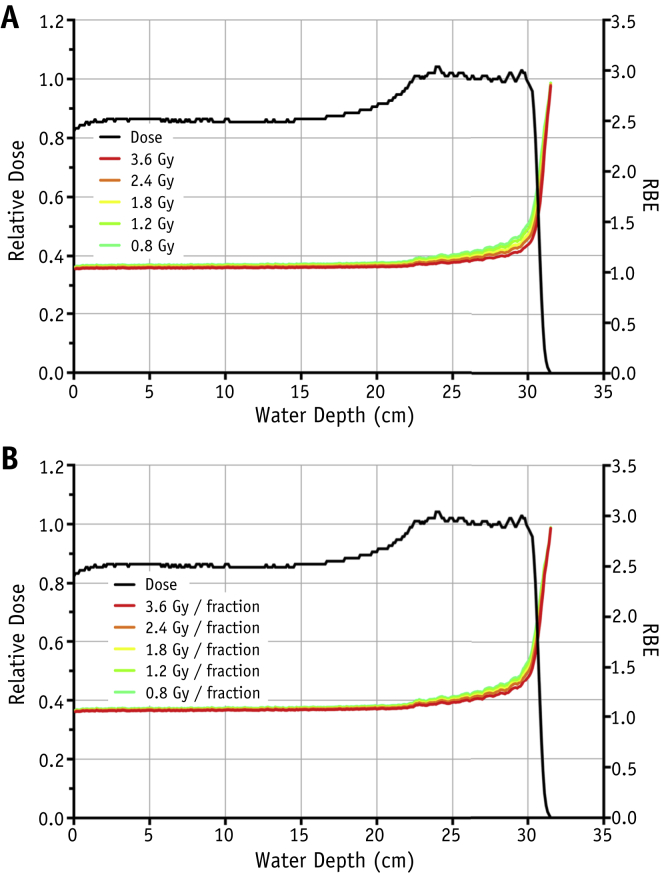
Parameterized relative biologically effective (RBE) values superimposed over spread-out Bragg peak dose-depth profiles for plateau doses of 3.6, 2.4, 1.8, and 0.8 Gy in acute (A) and fractionated (B) regimens. RBE values are calculated using equations (3), (4), (5) and X-ray data from Table E1 (available online at www.redjournal.org).

Parameterized RBE values as a function of proton dose and depth (ie, LET) for acute and fractionated regimens are shown in Figure 4. In agreement with literature data, RBE for acute exposure increases slowly in the SOBP region before rising sharply at the distal dose falloff. In the SOBP region there is also a small but significant increase in RBE as the total dose is reduced (from RBE = 1.12 for 3.6 Gy to RBE = 1.21 for 0.8 Gy). Under fractionated regimens, similar patterns for RBE with depth are observed, although the data indicate a smaller RBE increase in the SOBP region as a consequence of reducing the dose per fraction (from RBE = 1.17 for 3.6 Gy/fraction to RBE = 1.24 for 0.8 Gy/fraction).

**Fig. 5:**
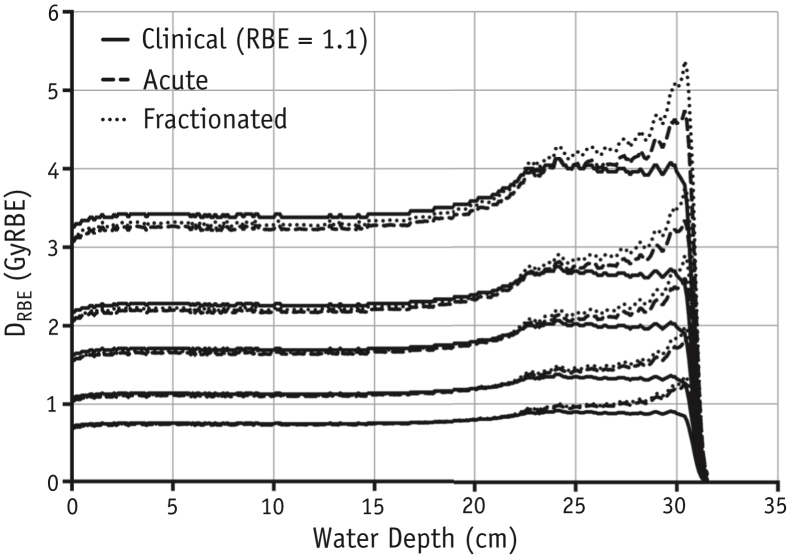
Spread-out Bragg peak (SOBP) relative biologically effective (RBE)-weighted absorbed dose (D_RBE_) profile comparing analytically obtained values when delivering an SOBP plateau dose of 3.6, 2.4, 1.8, and 0.8 Gy in both acute (dashed line) and fractionated (dotted line) regimens. Clinically assumed D_RBE_ values using a generic value of RBE = 1.1 are marked in solid line. RBE values obtained are for 225 kV_p_ X-ray doses of the same fraction size.

The clinical implication of these RBE increases for fractionated exposures is highlighted in Figure 5, where the experimental RBE-weighted absorbed dose D_RBE_ for acute and fractionated exposures for various dose sizes is presented alongside the clinically assumed profiles. Acute deliveries see significant increases in delivered D_RBE_ versus clinical assumptions (RBE = 1.1), particularly for smaller doses and in the distal region. Fractionation increases this effect in the SOBP region, seeing increases of 8.3% to 12.1% in integral D_RBE_ over the clinical case in comparison with 4.6% to 10.6% for the acute delivery of the same doses. The percentage increase is higher for smaller doses per fraction. The greatest difference between the experimental and clinically assumed D_RBE_ lies in the distal dose falloff region, as shown in Figure E4 (available online at www.redjournal.org). The increase in effective range (point of 80% peak D_RBE_) over the clinically assumed D_RBE_ profile shows a marked difference (∼7%) between acute and fractionated delivery toward higher SOBP doses (0.82 mm vs 0.88 mm at 3.6 Gy) (Fig. E5; available online at www.redjournal.org) before converging at lower doses per fraction.

### EQD2 of fractionated proton regimens

**Fig. 6:**
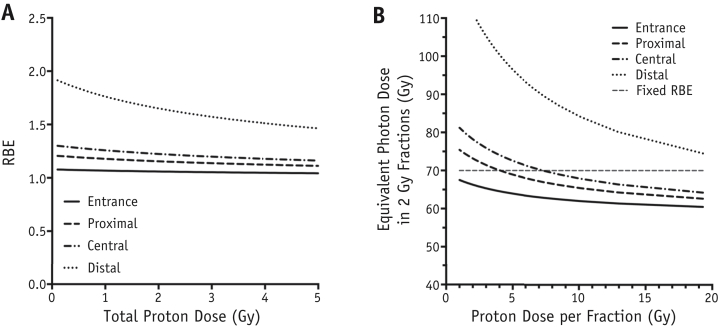
(A) Measurement of cell killing RBE relative to 225 kV_p_ X-rays as a function of acute proton dose at the 4 experimental positions on the SOBP. (B) Dose per fraction required to deliver a regimen equieffective to the conventional delivery of 35 fractions of 2 Gy X-rays. All data derived from proton α and β values obtained by the linear quadratic model.

The parameterized RBE for single fractions of proton radiation is shown in Figure 6A. Consistent with the sharp rise in LET, the highest RBE is found toward the more distal positions of the SOBP. For each position, RBE is higher for lower doses. The nonuniformity in biological effect when using a variable RBE is compounded under multifraction regimens, under low dose per fraction and in the low-dose regions of the proton dose profile in particular. As a result, it is useful to quantify the impact of a variable RBE on a typical clinical fractionation schedule.

Figure 6B outlines the equivalent photon dose in 2-Gy fractions (EQD2) for regimens considered isoeffective using a constant RBE of 1.1 to deliver a clinically relevant EQD2_1.1_ of 70 Gy to AG01522 cells. Details for EQD2 calculations are outlined in Supplementary Information (available online at www.redjournal.org). By incorporating a variable RBE using the same regimens, the predicted equivalent doses in all experimental positions deviate from those using the clinical assumption with an increase in predicted equivalent dose in hyperfractionated regimens and a reduction in the case of hypofractionated regimens. Notably, equivalent doses in the distal position are underestimated clinically for all fraction sizes.

## Discussion

The lack of robust experimental data exploring fractionated proton radiation presents a substantial opportunity to gain insight into the effects of an inhomogeneous cell response along clinical dose profiles. Although the sole use of the AG01522 cell line is not a comprehensive representation, this study provides useful reference data and highlights an interesting trend of RBE as a function of LET and fraction size, examining also the potential clinical implications. The findings from this report indicate a significant increase in RBE over the acute delivery of protons, where the same total physical doses are delivered in fractionated regimens. This is particularly evident toward the distal dose falloff (Fig. 5), where high RBE values have been reported for acute exposures 21, 22, 23, 24. Such an increase in effectiveness of fractionated exposures is proportional to the LET and inversely proportional to the dose per fraction delivered. Previously reported *in vivo* experiments 25, 26 have indicated a constant RBE with fractions for the middle of the SOBP but have acknowledged that the end of the SOBP was ∼1.14 more effective also for fractionated exposures. The data presented further highlight the inadequacy of extrapolating the cell response from X-ray radiation in the form of a generic, fixed RBE value of 1.1 and outline the difficulties in delivering isoeffective doses to treatment regions in terms of biologically effective beam range and dose.

Exposures to low-LET regions of the SOBP appear to produce cell survival levels similar to those of X-rays, with steeper and more linear survival curves correlating strongly with the increasing LET toward more distal regions. This increase in RBE with LET supports the hypothesis of more complex damage and has been observed in several previous studies for various cell lines 27, 28, and *in vivo* mouse models 25, 26 with the same linear relationship between α_p_ and LET having been previously reported through extensive analysis of current radiobiological data by Wedenberg *et al* (29).

The adoption of a linear quadratic formalism has successfully been used for clinical schedules using low LET radiation and closely describes the experimental data (30). The use of an experimental interfractional rest period of 24 hours reflects current clinical practice and appears to be adequate for the complete repair for sublethal damage for the AG01522 normal fibroblast cells. Complete repair of the fibroblast cells under these experimental conditions (eg, dose, LET) has previously been observed by the authors using immunofluorescence techniques to investigate DNA damage (31). Cells associated with more erroneous repair or exposure to higher LET radiation may promote incomplete repair between fractions, compounding sublethal repair to see higher than expected toxicity (32). The applicability of this approach for the full proton LET range and to tumor cells presents an avenue for further investigation.

Given the perception of protons as “low LET” radiation, there is a natural motivation to alter proton deliveries based on clinical experience with photons, with an advantageous dose deposition profile providing an incentive to deliver higher doses per fraction. Isoeffect calculations in this study have, however, outlined how the strong dependence of RBE on the proton LET component must be taken into consideration, particularly when varying dose per fraction. Additionally, this variation in RBE must be noted when comparing photon and proton schedules in the evaluation of clinical trials. The AG01522 cells in this study (α/β = 6.4 Gy) provide an insight into the behavior of late-responding tissues under various fractionation schedules. The increased effectiveness in the distal region for hyperfractionated exposures may reduce the therapeutic benefits of fractionation by counteracting the differential response with rapidly growing tumors. Additionally, the movement toward hypofractionation sees the potential for overestimation of effective dose delivered under the assumption of a constant RBE for all but the most distal regions of the SOBP.

The inhomogeneous cell killing response observed across the SOBP, the further effect of the different fractionation regimens on biological effectiveness, the effective range increases in the order of 1 mm, and the lack of confidence in predicting isoeffective treatments show significant limitations in the use of a generic, fixed value of RBE of 1.1. The experimental RBE variations and their implications for fractionated proton radiation therapy observed in this study support the incorporation of a variable RBE in the planning of clinical treatments. This study provides a dataset from primary human cells that can be used for assessing optimization strategies for fractionated proton radiation therapy in line with similar studies on variable RBE in treatment planning (17).
