# Supplementary material for: Investigating the Implications of a Variable RBE on Proton Dose Fractionation Across a Clinical Pencil Beam Scanned Spread-Out Bragg Peak
Source: Int J Radiat Oncol Biol Phys. 2016 May 1;95(1):70–7. doi: 10.1016/j.ijrobp.2016.02.029 (PMC4838672; doi:10.1016/j.ijrobp.2016.02.029)
Supplement: Supplementary Material [file mmc1.docx]

**Supplementary Information.**

**Depth dose and LET profile**

For all irradiations the Entrance position received 86% of the plateau dose with the Proximal, Centre and Distal positions located at the beginning, middle and end regions of the SOBP plateau each receiving 100% of the prescribed dose to a tolerance of <5% in all cases. In **Supplementary Figure 1** it is evident how LET remains relatively low for the initial positions, rising significantly towards the end of the ion path and at the Distal experimental position. LET in the Entrance, Proximal, Centre and Distal SOBP positions were determined through Monte Carlo simulations to be 0.630 ± 0.010, 1.680 ± 0.010, 2.45 ± 0.06 and 7.5 ± 0.7 keV/µm respectively. A dose averaged LET profile was simulated in a pure water phantom with voxels of size 1 mm. For a particle depositing an energy ΔE over a step length Δx, the dose average LET in the voxel was given by –totalLetD[voxel] / DtotalLetD[voxel], where totalLetD[voxel] = ΔE*(ΔE/Δx) summed over all particles in voxel, and DtotalLetD[voxel] = ΔE summed over all particles in voxel. The default step size for particle transport was set to 1 m, a value sufficiently large to ensure no artificial step limitation took place. Secondary particles produced were transported when their energy corresponded to a range of 0.05 mm. Such values allowed practical calculation times while providing reliable LETd values (1, 2) while the grouping energy losses and lengths of all steps during proton transport within each voxel ensured the reduction of high LET components due to boundary effects (3). The position of each cell monolayer on the LET/Dose profile was determined through conversion of virtual water (RW3, density – 1.022 g/cm^3^), polystyrene (density – 1.05 g/cm^3^) and cell culture media (density – 1.0 g/cm^3^) thicknesses to water equivalent depths through relative density calculations. The dose and simulated LET profile of the experimental SOBP is presented in Supplementary Figure 1.

**Irradiation**

A series of four media filled T25 cell culture flasks positioned at various water equivalent depths (achieved using solid plastic slabs) were irradiated with the cell monolayer (in exponential growth) positioned perpendicular to the horizontal proton beam. The irradiation of the volume 10 cm x 16 cm x 8 cm was performed using layers composed by monoenergetic proton spots with a spot spacing of 2 mm. The size of the monoenergetic proton layer was 10 x 16 cm^2^ to ensure that the whole sample was placed in the beam, even if there were positioning uncertainties. The dose in the samples was calculated using the dose calibration and the depth-dose curves used for the construction of the proton beam model used in the Proton therapy Centre in Prague treatment planning system for the pencil beam scanning mode. The different places in the SOBP where simulated using RW3 (1.022 water equivalent thickness) plates (PTW, Freiburg, Germany). The dose calibration for the treatment planning system was provided using a PPC05 ionization chamber (IBA Dosimetry) at 2 cm of water in a water tank. The number of monitor units required to reach the desired dose of 1.2, 0.8, 0.6 and 0.3 Gy were then calculated. The agreement of the calculated and the measured dose was within 1%. Moreover due to possible geometrical uncertainties in sample positioning, the total dose deviation does not exceed 2%.

Post irradiation, cells were trypsinized, counted and re-seeded at the appropriate density into six-multiwell plates. The number of cells seeded per well of the six-well plate was adjusted based on the absorbed dose with the aim of obtaining approximately 50 colonies/well. For example, for a predicted SF = 50% and considering a colony forming efficiency of 10% a total of 1000 cells was plated with the aim of producing approximately 50 colonies. In the case of the lowest surviving fractions ~1%, the same method was applied with the aim of producing approximately 20 colonies (i.e., 20,000 cells seeded). Reference X-ray data was obtained by irradiating cell monolayers in horizontal T25 flasks under identical cell culture conditions to proton exposures. Cells were irradiated by 225 kVp X-rays hardened by a 2 mm copper filter in an X-RAD 225 cabinet irradiator (Precision X- ray Inc., USA) at a dose rate of 0.59 Gy/min. Calibration of the X-ray source was carried out using a secondary standard electrometer and chamber from the Cancer Centre at Centre for Cancer Research and Cell Biology, Queen’s University Belfast, UK. Survival data reported represent the average of two independent experiments; error bars have been calculated by propagating the standard error of the mean for each multiwell plate.

**Simulation**

Representative dose- and dose averaged LET-depth profiles were obtained using the Geant4 Monte Carlo toolkit (1–4) whereby a water equivalent phantom of dimensions identical to the experimental setup was irradiated with an array of proton beamlets. The beamlet properties required to form the experimental SOBP such as energy, dimensions and weighting were obtained from the clinical treatment planning software used during sample irradiations.

**EQD2 Calculations**

Assuming a generic RBE of 1.1, the equivalent photon dose delivered in 2 Gy fractions for protons can be described by Equation 3, as derived by Dasu *et al.* (5):

EQD2_1.1_= $1.1 n_{prot}d_{prot}\frac{\left( 1 +\frac{1.1d_{prot}}{\left( \frac{\alpha_{x}}{\beta_{x}} \right)} \right)}{\left( 1 + \frac{2 Gy}{\left( \frac{\alpha_{x}}{\beta_{x}} \right)} \right)}$ (5)

where n_proton_ and d_proton_ represent the total number of fractions and dose per fraction for proton exposures respectively, α_x_ and β_x_ represent the linear-quadratic parameters for X-ray exposures. Conversely, using a similar formalism the variation in the biological effectiveness of protons can be accounted for by the use of a Dose Modifying Factor (DMF) where DMF_α_ = (α_p_ / α_x_) and DMF_β_ = 1 under the parameterized behaviors of α_p_ and β_p_ with LET.

EQD2_vari_= $n_{prot}d_{prot}\frac{\left( {DMF}_{\alpha} +\frac{{DMF}_{\beta} d_{prot}}{\left( \frac{\alpha_{x}}{\beta_{x}} \right)} \right)}{\left( 1 + \frac{2 Gy}{\left( \frac{\alpha_{x}}{\beta_{x}} \right)} \right)}$ (6)

**Dose Volume Histograms**

Dose Volume Histograms (DVH) of the full proton profile were analyzed over several regions of interest. Alongside analysis of the full proton path, the entrance region (from phantom entrance to up to 90% plateau dose), plateau region (90% - 90% plateau dose) and distal dose fall off (DDF) region (beyond 90% plateau dose) were considered separately. For the calculation of integral BED increases, the entrance and plateau regions were considered as the “SOBP region”.


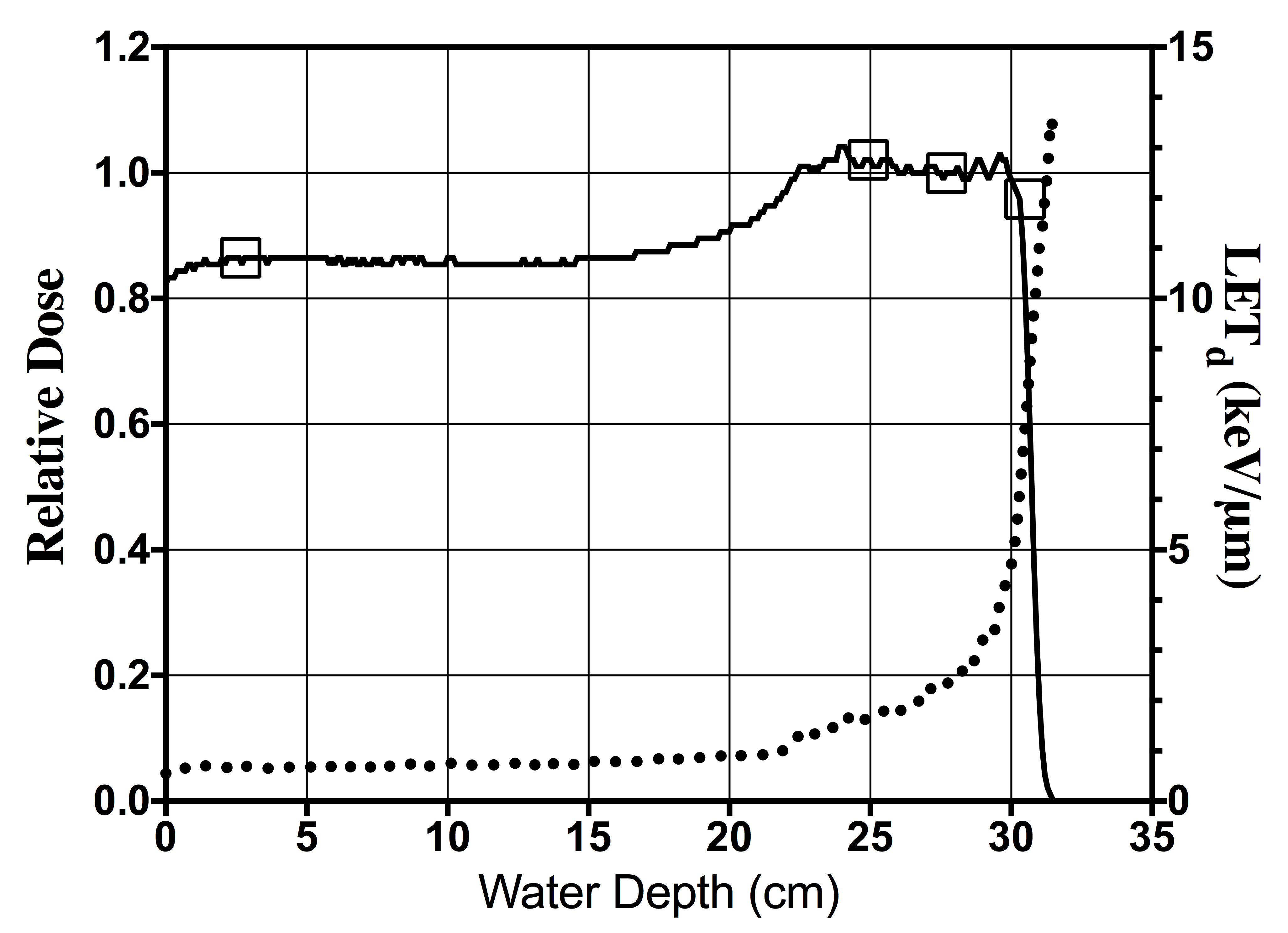


**Supplementary Figure 1.** Dose and dose averaged LET profiles (LET_d_) for actively scanned modulated proton beam with maximum energy 219.65 MeV. Vertical lines mark the four cell irradiation positions at the Entrance, Proximal, Centre and Distal positions. Relative dose and GEANT4 derived dose averaged LET values are indicated in dashed and solid black lines respectively.

**Supplementary Table 1.** Experimental positions at which AG01522 cells were exposed on the actively scanned proton SOBP with maximum energy 219.65 MeV and X-rays (225 kVp). Dose averaged LET values are simulated using the Geant4 Monte Carlo toolkit. Linear quadratic parameters alpha and beta for single, double and triple exposures with 24 hours between fractions are quoted alongside the standard error calculated using Graphpad Prism.

| Position | Depth (mm) | LET (keV/μm) | α(Gy^-1^) _single_ | β(Gy^-2^) _single_ | α(Gy^-1^) _double_ | β(Gy^-2^) _double_ | α(Gy^-1^) _triple_ | β(Gy^-2^) _triple_ |
| --- | --- | --- | --- | --- | --- | --- | --- | --- |
| Entrance | 26.6 | 0.630 ± 0.010 | 0.660 ± 0.079 | 0.101 ± 0.062 | 0.293 ± 0.031 | 0.042 ± 0.022 | 0.236 ± 0.007 | 0.007 ± 0.004 |
| Proximal | 249.3 | 1.680 ± 0.010 | 0.723 ± 0.024 | 0.1261 ± 0.0197 | 0.327 ± 0.049 | 0.061 ± 0.034 | 0.304 ± 0.011 | - |
| Centre | 277.1 | 2.45 ± 0.06 | 0.798 ± 0.022 | 0.156 ± 0.019 | 0.380 ± 0.018 | 0.061 ± 0.013 | 0.271 ± 0.004 | 0.022 ± 0.003 |
| Distal | 304.9 | 7.5 ± 0.7 | 1.253 ±  0.133 | 0.096 ± 0.139 | 0.709 ± 0.011 | - | 0.416 ± 0.007 | 0.030 ± 0.006 |
|  |  |  |  |  |  |  |  |  |
| X-ray | - | - | 0.635 ± 0.047 | 0.100 ± 0.034 | 0.257 ± 0.015 | 0.027 ± 0.009 | 0.210 ± 0.002 | 0.006 ± 0.001 |

**Supplementary Figure 2.** Analytically obtained survival fraction values based on a repeated acute response using a linear-quadratic formalism vs. experimental survival values for AG01522 cells. Lines of best fit for single, double and triple exposures result in gradients of 1.023 – 1.074 with Pearson’s correlation coefficient of 0.98 – 0.97, corresponding to *p* < 0.0001. Dashed line represents Survival_Exp_ = Survival_Model._


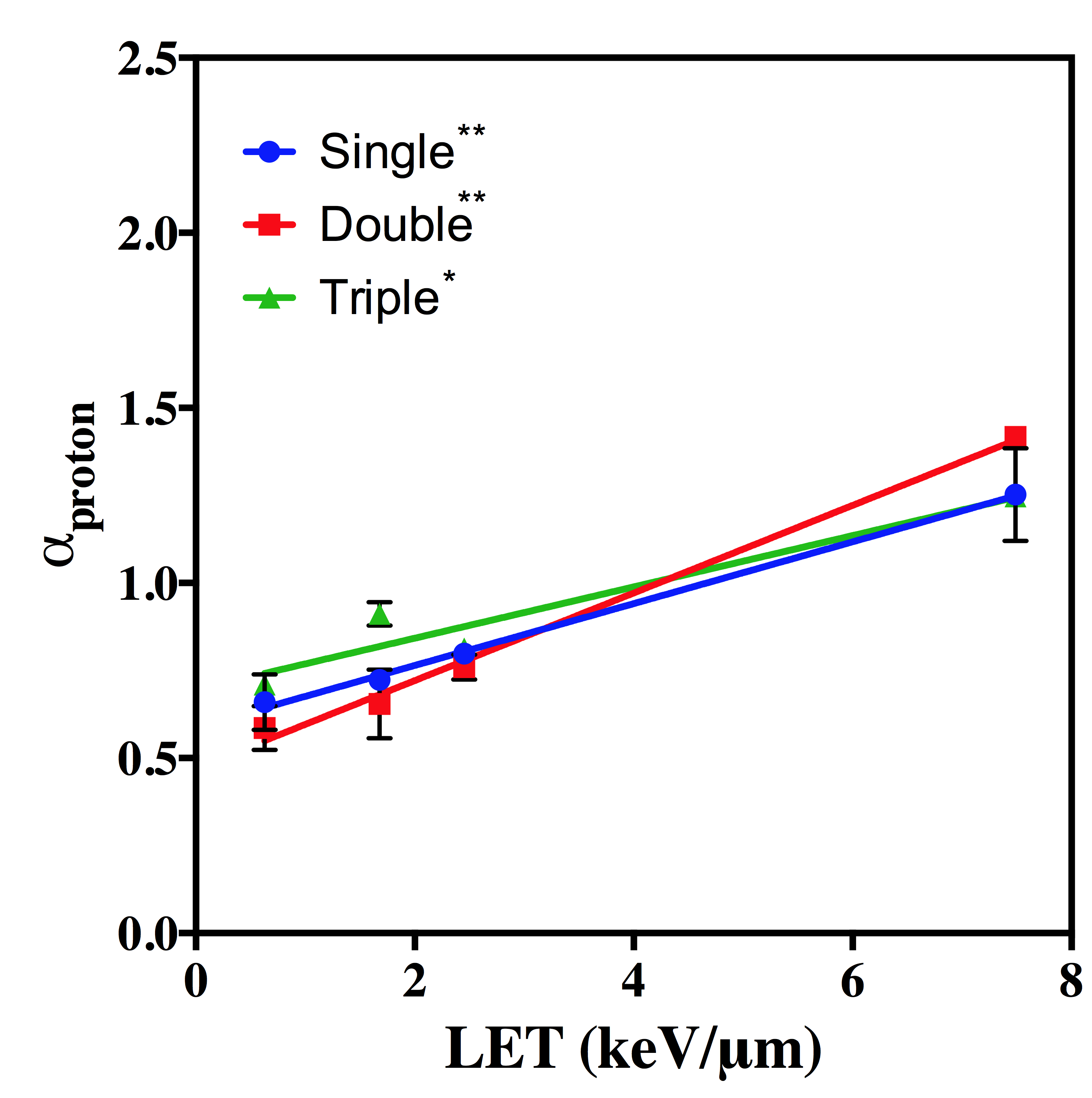

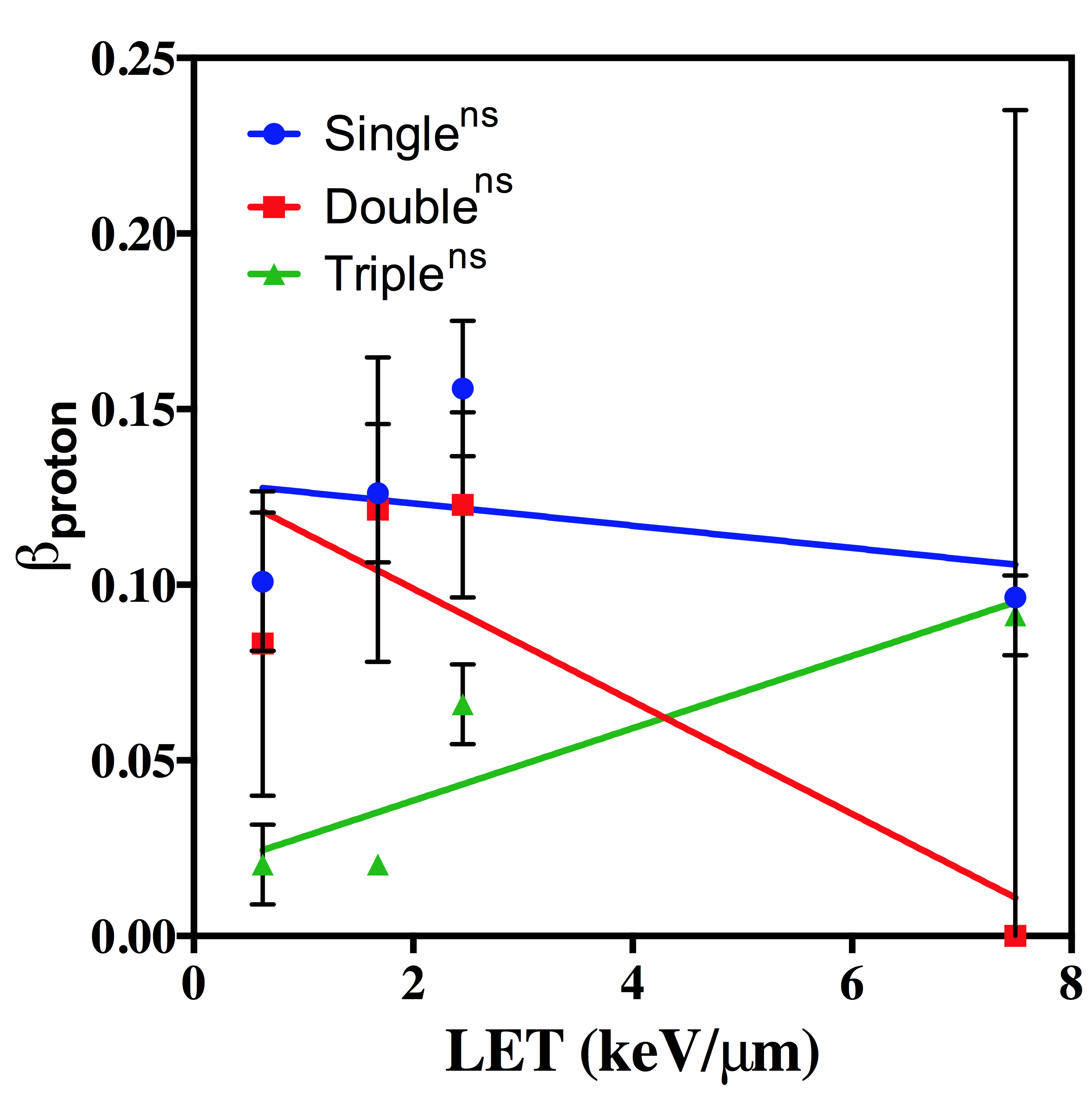


**Supplementary Figure 3.** Dependence of proton α and β parameters on LET for normal human fibroblast AG01522 cells. (A) Linear correlation of proton α values with LET. Linear regression analysis indicates R^2^ = 0.9978, 0.9946, 0.9168 for the single, double and triple exposures respectively where * indicates p < 0.05 and ** indicates p < 0.005. (B) Proton β values vs. LET where no correlation was found.


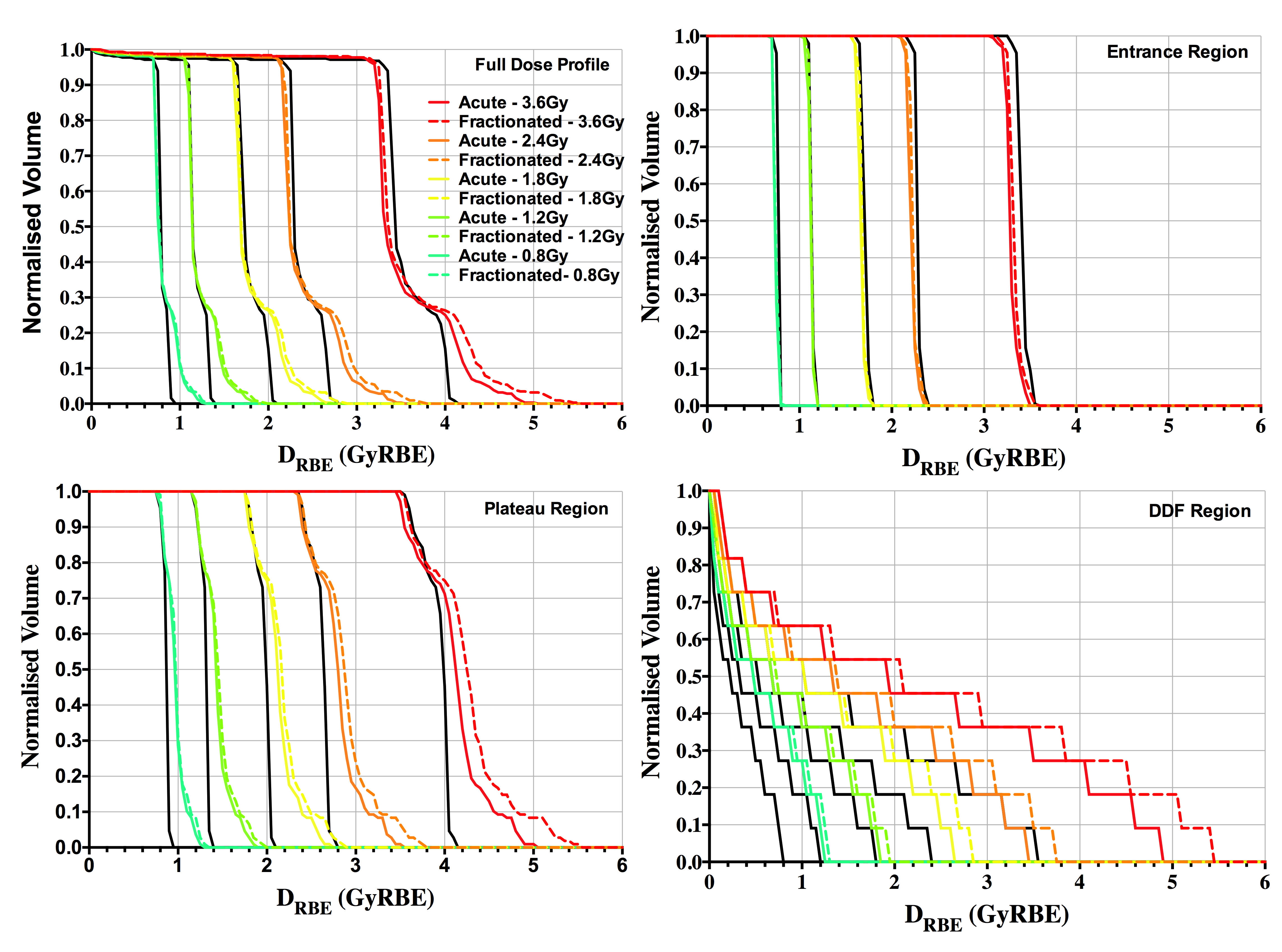


**Supplementary Figure 4**. Dose Volume Histograms (DVH) of the full proton D_RBE_ profile (A), the entrance region (up to 90% plateau dose, B), plateau region (90% - 90% plateau dose, C) and distal dose falloff region (beyond 90% plateau dose, D). Solid black lines indicate clinical assumption where RBE = 1.1, solid colour indicates D_RBE_ calculated using acute analytical RBE values and dashed colour indicates D_RBE_ calculated using fractionated analytical RBE. Noted doses indicated plateau doses.

**Supplementary Figure 5.** The increase in biologically effective range between doses delivered in acute (solid bars) and in fractionated regimes (open bars) versus the clinically assumed generic RBE obtained RBE weighted Dose (D_RBE_). Effective range is determined by the position of 80% peak D_RBE_ in the distal region.

|  | 50% SF | | | | | |
| --- | --- | --- | --- | --- | --- | --- |
|  | Single | | Double | | Triple | |
|  | **RBE** | RBE Err | **RBE** | RBE Err | **RBE** | RBE Err |
| Entrance | **1.03** | 0.68 | **1.16** | 0.70 | **1.12** | 0.40 |
| Proximal | **1.13** | 0.74 | **1.32** | 0.80 | **1.40** | 0.50 |
| Central | **1.25** | 0.82 | **1.48** | 0.88 | **1.33** | 0.48 |
| Distal | **1.72** | 1.14 | **2.44** | 1.45 | **1.99** | 0.79 |
|  |  |  |  |  |  |  |
|  | 10% SF | | | | | |
|  | Single | | Double | | Triple | |
|  | **RBE** | RBE Err | **RBE** | RBE Err | **RBE** | RBE Err |
| Entrance | **1.02** | 0.65 | **1.18** | 0.68 | **1.11** | 0.39 |
| Proximal | **1.13** | 0.70 | **1.36** | 0.79 | **1.31** | 0.46 |
| Central | **1.25** | 0.77 | **1.48** | 0.83 | **1.40** | 0.49 |
| Distal | **1.40** | 0.88 | **2.04** | 1.14 | **2.01** | 0.77 |
|  |  |  |  |  |  |  |
|  | 1% SF | | | | | |
|  | Single | | Double | | Triple | |
|  | **RBE** | RBE Err | **RBE** | RBE Err | **RBE** | RBE Err |
| Entrance | **1.02** | 0.63 | **1.19** | 0.67 | **1.10** | 0.38 |
| Proximal | **1.13** | 0.67 | **1.39** | 0.79 | **1.22** | 0.41 |
| Central | **1.25** | 0.74 | **1.49** | 0.81 | **1.46** | 0.50 |
| Distal | **1.18** | 0.71 | **1.72** | 0.93 | **2.03** | 0.74 |

**Supplementary Table 2.** The experimental RBE values calculated using linear-quadratic fitting parameters quoted in Supplementary Table 1.

**References**

1. Granville DA, Sawakuchi GO. Comparison of linear energy transfer scoring techniques in Monte Carlo simulations of proton beams. Phys. Med. Biol. 2015;60:N283–N291.

2. Guan F, Peeler C, Bronk L, et al. Analysis of the track- and dose-averaged LET and LET spectra in proton therapy using the geant4 Monte Carlo code. Med. Phys. 2015;42:6234–6247.

3. Cortés-Giraldo MA, Carabe A. A critical study of different Monte Carlo scoring methods of dose average linear-energy-transfer maps calculated in voxelized geometries irradiated with clinical proton beams. *Phys. Med. Biol.* 2015;60:2645–2669

4. Agostinelli S, Allison J, Amako K, *et al.* Geant4—a simulation toolkit. *Nucl. Instruments Methods Phys. Res. Sect. A Accel. Spectrometers, Detect. Assoc. Equip.* 2003;506:250–303.

5. Dasu A, Toma-Dasu I. Impact of variable RBE on proton fractionation. *Med. Phys.* 2013;40:011705.

6.Allison J, Amako K, Apostolakis J, *et al.* Geant4 Developments and Applications *IEEE TRANSACTIONS ON NUCLEAR SCIENCE*, VOL. 53, NO. 1, FEBRUARY 2006;53:270–278.

7. Cirrone G.A.P., Cuttone G, Mazzaglia SE, *et al.* Hadrontherapy : a Geant4-Based Tool for Proton / Ion-Therapy Studies. 2011;2:207–212.

8. Cirrone G.A.P, Cuttone G, Di Rosa F, *et al.* Hadrontherapy: An open source, Geant4-based application for proton-ion therapy studies. In: *IEEE Nuclear Science Symposium Conference Record*. IEEE; 2009:4186–4189.
